# Physicians Visiting List

**Published:** 1874-01

**Authors:** 


*The Physicians' Visiting List for* 1874. Philadelphia: Lindsay & Blakiston.

The Visiting List of Messrs. Lindsay and Blakiston, has been so long known by the profession, that should its publication be discontinued they would feel that they had sustained a severe loss. It makes its appearance this year in its usual form, and requires of us no formal introduction to the Profession. We have long considered it the neatest and most simple visiting list for physicians published.

